# Prospective multi-center study of oncologic outcomes of robot-assisted partial nephrectomy for pT1 renal cell carcinoma

**DOI:** 10.1186/1471-2490-12-11

**Published:** 2012-04-30

**Authors:** Rachel L Kyllo, Youssef S Tanagho, Jihad H Kaouk, Michael D Stifelman, Craig G Rogers, Shahab P Hillyer, Shyam S Sukumar, Kenneth G Nepple, Sam B Bhayani

**Affiliations:** 1 Division of Urology, Washington University School of Medicine, St Louis, MO, USA; 2 Glickman Urological Institute, Cleveland Clinic, Cleveland, OH, USA; 3 Department of Urology, NYU Langone School of Medicine, New York, NY, USA; 4 Department of Urology, Henry Ford Hospital, Detroit, MI, USA; 5 Division of Urology, Washington University School of Medicine, 660 S. Euclid Avenue, St. Louis, MO, 63110, USA

**Keywords:** Renal cell carcinoma, Partial nephrectomy, Robotic partial nephrectomy, Oncologic outcomes, Nephron-sparing surgery

## Abstract

**Background:**

Partial nephrectomy has been increasingly recommended over radical nephrectomy for the management of small renal masses based on improved renal functional outcomes without sacrifice of oncologic effectiveness. Robot-assisted partial nephrectomy (RAPN) has been introduced in an effort to offer another minimally invasive option for nephron-sparing surgery. However, reports of RAPN have been limited to short-term perioperative outcomes. The goal of this study is to report and evaluate the initial oncologic outcomes of RAPN. Utilizing prospectively obtained data on RAPN performed by four surgeons at four separate tertiary care centers, we selected patients with unilateral, localized, non-familial, pathologically-confirmed pT1 renal cell carcinoma and a minimum post-operative follow-up of 12 months.

**Methods:**

Utilizing prospectively obtained data on RAPN performed by four surgeons at four separate tertiary care centers, we selected patients with unilateral, localized, non-familial, pathologically-confirmed pT1 renal cell carcinoma and a minimum post-operative follow-up of 12 months. Survival analysis (disease-free, cancer-specific, and overall survival) was performed, and Kaplan-Meier curves were generated.

**Results:**

RAPN was performed in 124 patients with a median tumor size of 3.0 cm (IQR 2.2-4.2 cm). Median follow-up was 29 months (range 12-46 months). Positive parenchymal surgical margins occurred in two patients (1.6 %), both of whom were recurrence-free at 30 and 34 months after surgery. The three-year Kaplan-Meier estimated disease-free survival was 94.9 %, cancer-specific survival was 99.1 %, and overall survival was 97.3 %.

**Conclusions:**

In our cohort of patients with small renal carcinomas who were followed for a median of 29 months, recurrence and survival outcomes were similar to those reported for open and laparoscopic partial nephrectomy. Further long-term outcomes will be needed to definitively claim that RAPN is oncologically equivalent to other surgical approaches.

## Background

The incidence of small renal neoplasia is increasing, likely secondary to the increasing use of cross-sectional imaging [1]. Although, traditionally, radical nephrectomy has been performed for the definitive treatment of suspected small renal cell cancers, partial nephrectomy has become increasingly popular as a nephron-sparing option [2]. The American Urological Association’s guidelines for management of the clinical T1 renal mass place partial nephrectomy as a standard treatment option [3].

Partial nephrectomy may be accomplished via a variety of techniques. Open partial nephrectomy (OPN) has been shown to have excellent long-term oncologic outcomes which are similar to radical nephrectomy [4,5]. Laparoscopic partial nephrectomy (LPN) was developed as a minimally invasive alternative to open partial nephrectomy, and studies have also shown cancer control comparable to OPN [6,7]. More recently, robot-assisted partial nephrectomy (RAPN) has been developed as another option for the excision of a small renal mass. Preliminary studies have shown an acceptable learning curve with feasible results [8-11]. Complications of the RAPN approach are appropriate (15.8% overall complication rate) [12], as are initial perioperative pathological outcomes (positive margin rate of 1.6% - 4.1%) [8,13-15]. We previously reported multi-institutional analyses of perioperative and functional outcomes of RAPN- including complication rates, blood loss, warm ischemia time, post-operative changes in glomerular filtration rate, and hospital stay- and demonstrated favorable outcomes in all of these parameters [8,15].

Despite the favorable perioperative results of RAPN, there are no current studies focused on survival outcomes of this newer procedure. Nascent procedures should undergo analysis and reporting of oncologic outcomes to ensure that survival curves are not inferior to those of established procedures. Previous reports of OPN and LPN show disease-free survival (DFS) rates of 83% - 98%, cancer-specific survival (CSS) rates of 82% - 99%, and overall survival (OS) rates of 72% - 96%, respectively [4-6,16-20]. A formal assessment of oncologic outcomes of RAPN is absolutely necessary to demonstrate comparative efficacy of the robotic approach. Therefore, the primary goal of this multi-center study is to examine the intermediate-term oncologic outcomes of pT1 renal cell cancers which have been treated with RAPN. The goal will be to assess an “oncologic checkpoint” to ensure that the outcomes of this procedure are not oncologically inferior to the excellent outcomes that have been reported with LPN and OPN.

## Results

Demographic and clinical data from the study group (n = 124) are presented in Table 1. Median patient age was 58 years, and median age-adjusted Charlson Comorbidity Index was 1. Table 2 demonstrates the pathological characteristics of the tumors and data on cancer outcomes. Median tumor size was 3.0 cm, and 107 (86.3%) were staged pT1a, with the remaining 17 (13.7%) staged as pT1b. Twelve (9.7%) tumors were classified as Fuhrman grade I, 65 (52.4%) as Fuhrman grade II, 30 (24.2%) as Fuhrman grade III, and one (0.8%) as Fuhrman grade IV; grade was not reported in 16 patients. The predominant RCC subtype was clear cell (66.9%), with a smaller proportion of papillary (21.0 %) and chromophobe (9.7%) subtypes. Positive parenchymal margins occurred in two cases (1.6%); both of these patients had pT1a RCC and were free of recurrence at 30 and 34 months of follow-up.

**Table 1:** Demographic and pre-operative patient characteristics in 124 patients undergoing RAPN

**No. Patients**	**124**
Median Age (IQR) years	58.0 (52.6-67.6)
Median months follow-up (IQR)	29.0 (25.1-34.0)
No. Sex (%)	
Male	80 (64.5)
Female	44 (35.5)
No. Race (%)	
Caucasian	100 (80.6)
African American	14 (11.3)
Other	10 (8.1)
Median ASA classification (IQR)	2 (2-3)
Median Charlson Comorbidity Index (IQR)	1 (0-2)
Median BMI (IQR) kg/m^2^	28.3 (25.1-33.6)

**Table 2:** Tumor characteristics and oncologic outcomes in 124 patients undergoing RAPN

**Median cm Pathological Size (IQR)**	**3.0 (2.2-4.2)**
No. Side (%)	
Left	63 (50.8)
Right	61 (49.2)
No. Histology (%)	
Clear	83 (66.9)
Papillary	26 (21.0)
Chromophobe	12 (9.7)
Other	3 (2.4)
No. Fuhrman Grade (%)	
I	12 (9.7)
II	65 (52.4)
III	30 (24.2)
IV	1 (0.8)
Unavailable	16 (12.9)
No. Pathological Stage (%)	
T1a	107 (86.3)
T1b	17 (13.7)
No. Positive Margins (%)	2 (1.6)
No. Renal Recurrences (%)	1 (0.8) - ipsilateral
No. Distant Recurrences (%)	1 (0.8)

Median follow-up was 29.0 months (IQR 25.1-34.0 months, total range 12-46 months). Two patients (1.6%) had recurrence of renal cell cancer at 16 and 35 months post-RAPN (details described below). One of the recurrences was in the ipsilateral kidney, and the other had widespread intraperitoneal metastases. Both patients with recurrence had negative surgical margins on their original RAPN specimens.

The one ipsilateral recurrence occurred in a patient with pT1a, Fuhrman grade II clear RCC. A completely endophytic 1.7 cm hypervascular renal lesion developed 35 months after RPN; this recurrence was treated with percutaneous cryoablation. No diagnostic biopsy of the recurrence was obtained, but this event is coded as a recurrence for the purposes of this communication. The second recurrence was a presumed metastatic renal cell cancer. This patient originally had a 2.7 cm pT1a, Fuhrman grade II papillary RCC excised with negative margins. The patient developed metastases (nodal, solid organ, and omental studding) 16 months after RAPN. Tissue diagnosis of the metastases was not definitive, but renal cell cancer was the only confirmed malignancy in this patient. This patient expired seven months after metastasis development (23 months after the original RAPN) and is coded as a cancer-specific mortality for this study, although an alternative secondary malignancy cannot be ruled out. There was no evidence of tumor spillage, seeding, or known divergence from oncologic standards during the patient’s original RAPN procedure, and explanation of the pathophysiology of this recurrence is not possible.

Death from other non-cancer causes occurred in three individuals. One patient experienced a cardiovascular-related morbidity 24 months after surgery. A second patient expired from metastatic breast cancer 43 months after RAPN. The last morbidity was a death from an unknown cause 22 months after surgery; however, this patient was disease-free on imaging performed prior to expiration.

**Figure 1:**
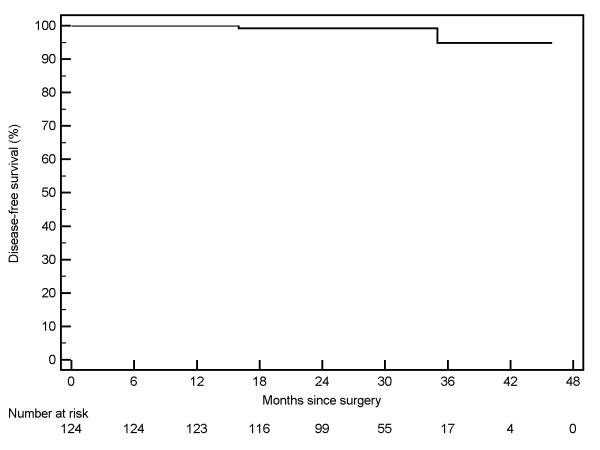
**Disease free survival of 124 patients undergoing robotic partial nephrectomy.**

**Figure 2:**
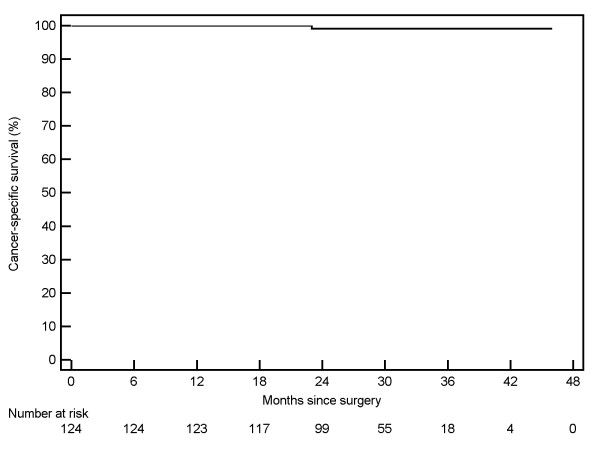
Cancer-specific survival of 124 patients undergoing robotic partial nephrectomy.

**Figure 3:**
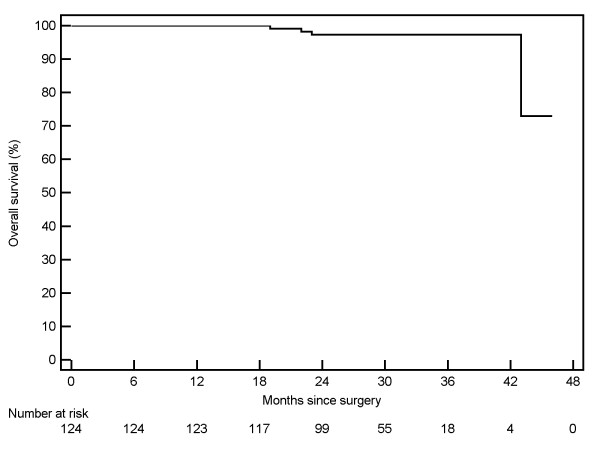
Overall survival of 124 patients undergoing robotic partial nephrectomy.

Kaplan-Meier curves were generated for 124 patients with pT1 RCC. The three-year Kaplan-Meier estimated disease-free survival [Figure 1] rate was 94.9% (95% CI: 86.5% - 100%). The three-year cancer-specific survival [Figure 2] was estimated at 99.1% (95% CI: 97.3 - 100%), and the overall survival rate was estimated at 97.3% (95% CI: 94.3% - 100%) [Figure 3].

## Discussion

Nephron-sparing surgery (NSS) has emerged as a standard of care for treating localized renal malignancy [3]. Numerous studies have demonstrated that NSS is a safe and effective alternative to radical nephrectomy for patients with small renal tumors, and the use of NSS has been associated with improved renal functional outcomes and significant decreases in cardiovascular disease and overall mortality [21,22].

Open partial nephrectomy is a well-established procedure with studies showing similar oncologic outcomes to radical nephrectomy. Laparoscopic partial nephrectomy has also been studied with similarly satisfactory oncologic outcomes. Robot-assisted partial nephrectomy has not been studied with regards to intermediate-term survival outcomes, as the procedure was first reported in 2004 and did not appear in increasing numbers in the literature until 2008 [23]. As such, the existing literature on RAPN has focused on feasibility, perioperative outcomes, and surgical margin status. As with all new oncologic procedures, it is critical to report early outcomes to ensure that survival curves are appropriate relative to established procedures. In this multi-center study with a median follow-up of 2.5 years and a maximum follow-up of nearly four years, we report intermediate-term oncologic outcomes similar to those that have been previously published for LPN and OPN.

Table 3 outlines survival outcomes of OPN and LPN from several recent studies reported in the literature. The three-year DFS herein for pT1 (a and b) lesions (94.9%) is similar to those previously reported for OPN and LPN (84% - 97.6%). Other papers have not reported DFS, but instead documented metastasis-free survival at 83% – 98%. RAPN in this study shows a three-year Kaplan-Meier estimated CSS of 99.1% at a median follow-up of 29months. This compares to other studies of OPN and LPN, which have reported CSS at three years or greater of 82 % - 99% and 93% - 99.3%, respectively. Additionally, the three-year OS herein (97.3%) is comparable to those demonstrated in the literature for OPN and LPN (Table 3).

**Table 3:** *Reported oncologic outcomes of open (OPN) and laparoscopic partial nephrectomy (LPN).* MFS = metastasis-free survival; DFS = disease-free survival; CSS = cancer-specific survival; OS = overall survival

**Study Name**	**Stages**	**N**	**Surgery**	**Follow-up (months)**	**MFS or DFS**	**CSS**	**OS**
**Lerner et al. 1996**^**15**^	Robson stage ≤ II	185	OPN	44.4 (mean)	83 % MFS @ 5y	89 % @ 5y	77 % @ 5y
**Hafez et al. 1999**^**5**^	T1, T2, T3a, T3b	485	OPN	47 (mean)	NM	82 %-99 % @ 5y	81 % @ 5y
**Belldegrun et al. 1999**^**16**^	All	146	OPN	74 (median)	NM	98 %	86 %
**Lau et al. 2000**^**4**^	All	164	OPN	40.8 (median)	98 % MFS @ 5y	98 % @ 5y	91 % @ 5y
**Permpongkosol et al. 2006**^**17**^	T1	85	LPN	40.4 (mean)	91.4 % DFS @ 5y	NM	93.75 % @ 5y
	T1	58	OPN	49.7 (mean)	97.6 % DFS @ 5y	NM	95.8 % @ 5y
**Gill et al. 2007**^**6**^	T1	514	LPN	14.4 (median)	NM	99.3 % @ 3y	NM
	T1	676	OPN	33.6 (median)	NM	99.2 % @ 3y	NM
**Joniau et al. 2009**^**18**^	T1b	67	OPN	40.2 (median)	84 % DFS @ 5y	99 % @ 5y	72 % @ 5y
**Lane et al. 2010**^**19**^	All	499	LPN	48 (median)	86-97 % MFS @ 5y	93-97 % @ 5y	NM
	All	762	OPN	68.4 (median)	97 % MFS @ 5y	97 %-99 % @ 5y	NM
**Present study**	T1	124	RAPN	29 (median)	94.9 % DFS @ 3y	99.1 % @ 3y	97.3 % @ 3y

The patterns of recurrence are not known yet for robotic partial nephrectomy. For open partial nephrectomy, the most common sites of metastasis include lymph nodes, lungs, contralateral kidney, bone, brain, and liver. In this series, there was one case of peritoneal implantation, but without pathological confirmation that this was renal cell, it is hard to make any firm conclusions. Nevertheless, we have included it as a cancer related mortality for the purposes of reporting. It will be interesting to monitor sites of recurrence in the future.

Thus, the RAPN results reported here appear to be generally comparable to those previously documented for OPN and LPN. Longer term follow-up will be necessary to ensure no divergence from the standards set by OPN and LPN. It is critical to note that a detailed statistical comparison of outcomes between series is not possible secondary to differences among studies in follow-up, pathology, tumor grades, RCC subtypes, and reported data. Additionally, since the majority of cases are less than 4 cm in this study, perhaps even active surveillance would have resulted in similar outcomes. Again, long term studies are needed.

Despite these reasonable outcomes, caution is advised in interpreting the results of this study. As the outcomes are still at less than four-year follow-up, broad conclusions cannot be made regarding long-term oncologic outcomes. Moreover, studies have shown that the risk of RCC recurrence is highest between three and five years post-surgery [24,25]. Thus, more follow-up will be necessary to further evaluate the long-term oncologic outcomes of RAPN. Secondly, the surgeons herein are all high-volume robotic renal surgeons practicing in tertiary-care academic medical centers, and the generalization of these results to lower volume surgeons is unknown. Prior reports have identified improved outcomes comparing high-volume to low-volume surgeons in some procedures [26,27]. Nevertheless, this same argument can be applied to most of the OPN and LPN outcomes literature. Another concern is that this study is a retrospective review of prospectively maintained databases, so there are likely inherent unrecognized selection biases that would not exist in a randomized study. Lastly, it is important for surgeons to consider that oncologic outcomes are only one factor in choosing a treatment modality; other issues such as complications, ease of performance, patient acceptance, and cost are important issues which can influence treatment decisions. There certainly is no detectable superiority of RAPN from a cancer control perspective. Nevertheless, RAPN appears to have appropriate survival outcomes in this study. Longer term follow-up is needed to ensure no divergence in survival from established standards. Additionally, caution is advised in the interpretation of any survival analysis, since a recent randomized study showed a slightly lower overall survival with partial nephrectomy compared to radical nephrectomy [28].

## Conclusions

This study adds to the literature by establishing the first multi-center evaluation of oncologic outcomes following RAPN. In patients with localized renal cell cancer measuring less than 4 cm (and perhaps 7 cm, but overall numbers are low), RAPN was found to have a low rate of positive surgical margins and disease-free, cancer-specific, and overall survival rates similar to those previously reported for other surgical approaches. Although this “oncologic checkpoint” for RAPN is promising on a preliminary basis, further follow-up will be needed to critically assess the long-term oncologic outcomes of RAPN.

## Methods

Prospective data were collected from databases approved by institutional review boards (IRB), and separate IRB approval was obtained for pooling the data for a multi-center study. Demographic, clinical, and follow-up data were collected prospectively on patients who underwent RAPN at four tertiary-care U.S. academic urological centers from June 2006 to June 2009. All patients who underwent RAPN for a pathologically confirmed pT1 renal cell carcinoma (RCC) with a minimum follow-up of 12 months were included. Patients with bilateral synchronous tumors, known familial cancer syndrome, or known metastatic disease were excluded. Hence, the study cohort was limited to small (<7 cm), sporadic, organ-confined RCC. Comorbid conditions were assessed with the American Association of Anesthesiologists Score (ASA) and the age-adjusted Charlson Comorbidity Index [29].

RAPN was performed using previously published techniques [30-32]. The oncologic follow-up protocol consisted of physical examination and cross-sectional imaging of the chest and abdomen at three to six months postoperatively and annually thereafter to rule out recurrence. Additional imaging was left to the discretion of the primary urologist or primary care physician. Survival outcomes were evaluated using Kaplan-Meier survival analysis. Statistical analysis was completed using SPSS® 15, Medcalc 11.4, and Microsoft Excel 2007.

### IRB approval and consent

Washington University School of Medicine Human Studies Committee, Cleveland Clinic Institutional Review Board, Institutional Review Board, NYU Langone Medical Center, Henry Ford Institutional Review Board.

NOTE: Consent was obtained from patients for participation in this study.

## Abbreviations

RCC, Renal cell carcinoma; NSS, Nephron-sparing surgery; RAPN, Robot-assisted partial nephrectomy; OPN, Open partial nephrectomy; LPN, Laparoscopic partial nephrectomy; DFS, Disease-free survival; CSS, Cancer-specific survival; OS, Overall survival; MFS, Metastasis-free survival.

## Competing interests

Rachel L. Kyllo, B.S.: No competing interests exist. Youssef S. Tanagho, M.D., M.P.H.: No competing interests exist. Jihad Kaouk, M.D.: Intuitive Surgical, Inc. Michael Stifelman, M.D.: Intuitive Surgical, Inc. Craig Rogers, M.D.: Intuitive Surgical, Inc. Shahab Hillyer, M.D.: No competing interests exist. Shyam Sukumar, M.D.: No competing interests exist. Kenneth G. Nepple, M.D.: No competing interests exist. Sam B. Bhayani, M.D.: No competing interests exist.

## Authors’ contributions

RLK: data acquisition, data analysis and interpretation, drafting of manuscript, YST: drafting of manuscript; data analysis and interpretation; critical revision of the manuscript, JK: data acquisition, critical revision of the manuscript, supervision, MS: data acquisition, critical revision of the manuscript, supervision, CR: data acquisition, critical revision of the manuscript, supervision, SH: data acquisition, critical revision of the manuscript, SS: data acquisition, critical revision of the manuscript, KGN: critical revision of the manuscript; data analysis and interpretation, SBB: conception and design, drafting of manuscript, data acquisition, critical revision of the manuscript, supervision. All authors read and approved the final manuscript.

## Pre-publication history

The pre-publication history for this paper can be accessed here:

http://www.biomedcentral.com/1471-2490/12/11/prepub

## References

[B1] Sun M Thuret R Abdollah F Age-adjusted incidence, mortality, and survival rates of stage-specific renal cell carcinoma in North America: a trend analysis Eur Urol 2011 59 135 141 10.1016/j.eururo.2010.10.029 21035250

[B2] Cooperberg MR Mallin K Kane CJ Treatment trends for stage I renal cell carcinoma J Urol 2011 186 394 399 10.1016/j.juro.2011.03.130 21679982

[B3] Campbell SC Novick AC Belldegrun A Guideline for management of the clinical T1 renal mass J Urol 2009 182 1271 1279 10.1016/j.juro.2009.07.004 19683266

[B4] Lau WK Blute ML Weaver AL Matched comparison of radical nephrectomy vs nephron-sparing surgery in patients with unilateral renal cell carcinoma and a normal contralateral kidney Mayo Clin Proc 2000 75 1236 1242 10.4065/75.12.1236 11126830

[B5] Hafez KS Fergany AF Novick AC Nephron sparing surgery for localized renal cell carcinoma: impact of tumor size on patient survival, tumor recurrence and TNM staging J Urol 1999 162 1930 1933 10.1016/S0022-5347(05)68071-8 10569540

[B6] Gill IS Kavoussi LR Lane BR Comparison of 1,800 laparoscopic and open partial nephrectomies for single renal tumors J Urol 2007 178 41 46 10.1016/j.juro.2007.03.038 17574056

[B7] Allaf ME Bhayani SB Rogers C Laparoscopic partial nephrectomy: evaluation of long-term oncological outcome J Urol 2004 172 871 873 10.1097/01.ju.0000134292.36152.fa 15310986

[B8] Benway BM Bhayani SB Rogers CG Robot-assisted partial nephrectomy: an international experience Eur Urol 2010 57 815 820 10.1016/j.eururo.2010.01.011 20116163

[B9] Haber GP White WM Crouzet S Robotic versus laparoscopic partial nephrectomy: single-surgeon matched cohort study of 150 patients Urology 2010 76 754 758 10.1016/j.urology.2010.03.058 20646744

[B10] Deane LA Lee HJ Box GN Robotic versus standard laparoscopic partial/wedge nephrectomy: a comparison of intraoperative and perioperative results from a single institution J Endourol 2008 22 947 952 10.1089/end.2007.0376 18397157

[B11] Mottrie A De Naeyer G Schatteman P Impact of the learning curve on perioperative outcomes in patients who underwent robotic partial nephrectomy for parenchymal renal tumours Eur Urol 2010 58 127 132 10.1016/j.eururo.2010.03.045 20399002

[B12] Spana G Haber GP Dulabon LM Complications after robotic partial nephrectomy at centers of excellence: multi-institutional analysis of 450 cases J Urol 2011 186 417 422 10.1016/j.juro.2011.03.127 21679980

[B13] Rogers CG Menon M Weise ES Robotic partial nephrectomy: a multi-institutional analysis J Robotic Surg 2008 2 141 143 10.1007/s11701-008-0098-2 27628250

[B14] Dulabon LM Kaouk JH Haber GP Multi-institutional analysis of robotic partial nephrectomy for hilar versus nonhilar lesions in 446 consecutive cases Eur Urol 2011 59 325 330 10.1016/j.eururo.2010.11.017 21144643

[B15] Benway BM Bhayani SB Rogers CG Robot assisted partial nephrectomy versus laparoscopic partial nephrectomy for renal tumors: a multi-institutional analysis of perioperative outcomes J Urol 2009 182 3 866 872 10.1016/j.juro.2009.05.037 19616229

[B16] Lerner SE Hawkins CE Blute ML Disease outcome in patients with low stage renal cell carcinoma treated with nephron sparing or radical surgery J Urol 2002 167 884-9; discussion 889-90 8618276

[B17] Belldegrun A Tsui KH deKernion JB Efficacy of nephron-sparing surgery for renal cell carcinoma: analysis based on the new 1997 tumor-node-metastasis staging system J Clin Oncol 1999 17 2868 2875 10561364 10.1200/JCO.1999.17.9.2868

[B18] Permpongkosol S Bagga S Romero FR Laparoscopic versus open partial nephrectomy for the treatment of pathological T1N0M0 renal cell carcinoma: a 5-year survival rate J Urol 2006 176 1984-8; discussion 1988-9 10.1016/j.juro.2006.07.033 17070227

[B19] Joniau S Vander Eeckt K, Srirangam SJ, et al: Outcome of nephron-sparing surgery for T1b renal cell carcinoma BJU Int 2009 103 1344 1348 10.1111/j.1464-410X.2008.08230.x 19040528

[B20] Lane BR Gill IS 7-Year oncological outcomes after laparoscopic and open partial nephrectomy J Urol 2010 183 473 479 10.1016/j.juro.2009.10.023 20006866

[B21] Huang WC Elkin EB Levey AS Partial nephrectomy versus radical nephrectomy in patients with small renal tumors–is there a difference in mortality and cardiovascular outcomes? J Urol 2009 181 55-61; discussion 61-2 10.1016/j.juro.2008.09.017 PMC2748741 19012918

[B22] Go AS Chertow GM Fan D Chronic kidney disease and the risks of death, cardiovascular events, and hospitalization N Engl J Med 2004 351 1296 1305 10.1056/NEJMoa041031 15385656

[B23] Gettman MT Blute ML Chow GK Robotic-assisted laparoscopic partial nephrectomy: technique and initial clinical experience with DaVinci robotic system Urology 2004 64 914 918 10.1016/j.urology.2004.06.049 15533477

[B24] Hafez KS Novick AC Campbell SC Patterns of tumor recurrence and guidelines for follow-up after nephron sparing surgery for sporadic renal cell carcinoma J Urol 1997 157 2067 2070 10.1016/S0022-5347(01)64675-5 9146581

[B25] Montie JE Follow-up after partial or total nephrectomy for renal cell carcinoma Urol Clin North Am 1994 21 589 592 7974891

[B26] Birkmeyer JD Stukel TA Siewers AE Surgeon volume and operative mortality in the United States N Engl J Med 2003 349 2117 2127 10.1056/NEJMsa035205 14645640

[B27] Vickers AJ Savage CJ Hruza M The surgical learning curve for laparoscopic radical prostatectomy: a retrospective cohort study Lancet Oncol 2009 10 475 480 10.1016/S1470-2045(09)70079-8 19342300 PMC2777762

[B28] Van Poppel H Da Pozzo L Albrecht W A Prospective, Randomised EORTC Intergroup Phase 3 Study Comparing the Oncologic Outcome of Elective Nephron-Sparing Surgery and Radical Nephrectomy for Low-Stage Renal Cell Carcinoma Eur Urol 2011 59 543 552 10.1016/j.eururo.2010.12.013 21186077

[B29] Hall WH Ramachandran R Narayan S An electronic application for rapidly calculating Charlson comorbidity score BMC Cancer 2004 4 94 10.1186/1471-2407-4-94 15610554 PMC545968

[B30] Benway BM Wang AJ Cabello JM Robotic partial nephrectomy with sliding-clip renorrhaphy: technique and outcomes Eur Urol 2009 55 592 599 10.1016/j.eururo.2008.12.028 19144457

[B31] Bhayani SB da Vinci robotic partial nephrectomy for renal cell carcinoma: an atlas of the four-arm technique J Robotic Surg 2007 1 279 285 10.1007/s11701-007-0055-5 PMC4247423 25484978

[B32] Patel MN Bhandari M Menon M Robotic-assisted partial nephrectomy BJU Int 2009 103 1296 1311 10.1111/j.1464-410X.2009.08584.x 19402830

